# Stigma and Discrimination During the Covid-19 Pandemic

**DOI:** 10.1192/j.eurpsy.2022.2258

**Published:** 2022-09-01

**Authors:** H. Marchean, A. Mihai

**Affiliations:** 1 Mureș County Clinical Hospital, Târgu Mureș, 2nd Psychiatry Clinic, Tg Mures, Romania; 2 George Emil Palade” University of Medicine, Pharmacy, Science and Technology, M4, Psychiatry Department, Tg Mures, Romania

**Keywords:** Covid-19, stigma, review, discrimination

## Abstract

**Introduction:**

A frequently discussed topic today, stigma and/or discrimination are social phenomena that, in the broader context of medical discourse and especially in the current epidemiological situation, Covid-19 pandemic, appear and need a detailed examination

**Objectives:**

This study aims are to examine the literature and to present the aforementioned phenomena, comparing them with the Link & Phelan stigma model and offering pros and cons for their congruence with the model.

**Methods:**

Literature analysis with searching words: stigma, discrimination, Covid-19, medical and especially psychiatric pathology, in Pubmed and Google scholar engine.

**Results:**

The studied 32 articles provided 4 stigmatized subgroups in the social context of the pandemic: that of patients and medical staff, that of comorbidities sufferers, that of stigmatized ethnic groups, and that of stigmatized races. These groups, stigmatized directly or by overlapping with the “actual” group, were studied in the most relevant PubMed articles, and evidence for the congruence of their stigma with the model was presented in this review.

**Conclusions:**

This work could also serve as a starting point for further study on combating stigma, improving the lives of our patients, colleagues affected by occupational exposure, and, finally, society at large

**Disclosure:**

No significant relationships.

